# Garlic (*Allium sativum*) and mushroom (*Agaricus bisporus*) powder: Investigation of performance, meat quality, serum profile lipid, and intestinal morphology in broilers

**DOI:** 10.1002/vms3.70031

**Published:** 2024-09-17

**Authors:** Fatemeh Aziz‐Aliabadi, Hadi Noruzi, Zeyad Kamal Imari

**Affiliations:** ^1^ Department of Animal Science Faculty of Agriculture Ferdowsi University of Mashhad Mashhad Iran; ^2^ Department of Animal Production Techniques Technical College of Al‐Musaib Al‐Furat Al‐Awsat Technical University AL‐Kufa Iraq

**Keywords:** broiler, body weight gain, meat quality, medicinal plants, serum lipid

## Abstract

**Background:**

With the ban on the use of antibiotics in poultry nutrition, the opinion of nutritionists turned to their alternatives. Garlic and mushroom are the two important phytobiotic compounds in poultry nutrition.

**Objectives:**

This experiment was done to investigate the effect of garlic powder (GP) and mushroom powder (MP) on the growth performance, meat quality, serum lipid profile, and intestinal morphology of broilers.

**Methods:**

Five hundred and seventy‐six one‐day‐old male Ross 308 broiler chicks were assigned to eight treatments with six replications based on a completely randomized design in a factorial arrangement of 4*2 with four levels of GP (0.00, 0.50, 1.00, 1.50%) and two levels of MP (0.00, 1.00%).

**Results:**

No significant effects of GP and MP on the performance were observed. With increasing levels of GP in the diets, the lightness and redness of breast meat decreased and increased, respectively (*p* < 0.05). The effect of increasing the amount of GP on the reduction of total cholesterol level was similar in the absence or presence of MP. With increasing levels of GP in the diets, the villus height (VH) and VH to crypt depth ratio (VH: CD) increased. The use of MP in the diets significantly increased VH and VH: CD (*p* < 0.05).

**Conclusion:**

The addition of GP and MP to the broilers’ diets did not have any negative effect on the performance. These pharmaceutic herbs improved intestinal morphology. In addition, increasing the level of GP amended the meat color and reduced the level of serum cholesterol.

## Graphical abstract

Increasing the level of dietary garlic powder, improved the meat color and intestinal morphology and decreased the serum cholesterol level.

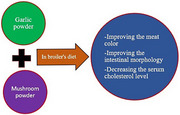

## 1 INTRODUCTION

The excessive and continuous use of antibiotics may cause adverse side effects such as suppression of the host's immune system and risks to the environment. Therefore, there is a growing interest in using their suitable alternatives such as plant feed additives (Kairalla et al., 2022a, 2022b, 2023). It has been shown the beneficial efficacy of a wide range of herbs on feed efficiency, antimicrobial, and immune‐stimulating in poultry and animal nutrition (Saleh et al., 2018). The use of phytogenic feed additives and natural yields comprising bioactive ingredients has shown a hopeful attitude in the poultry industry (Alagawany et al., 2019; Khafaga et al., 2019; Reda et al., 2019; Selim et al., 2021). Garlic (Allium sativum) has been used as a pharmaceutical herb for more than 4000 years (Corzo‐Matinez et al., 2007). Antihypertensive, antimicrobial and antioxidant properties of garlic have been proved in various studies (Chen et al., 2008; Kairallaet al., 2022b). The advantages associated with garlic utilization are ascribed to thiosulfonates, the most plentiful class of organic sulfur compounds (Lawson & Wang, 2001). Allicin usually accounts for mor than 70% of all thiosulfinates (Noruzi & Aziz‐Aliabadi, 2024). The mushroom (Agaricus bisporus) and its various derivatives encompass a large number of active materials such as ergothioneine, phenolic antioxidants, varigatic acid and dibiviquinone (Dubost et al., 2007; Kasuga et al., 1995). The effective substances of mushrooms have antioxidant, antibacterial, immune boosting, and stress‐reducing functions (Dalloul et al., 2006). Kansal et al. (2017) and Dalloul & Lillehoj (2006) stated that the use of garlic (0.75%) in the broilers’ diet would have a beneficial impact on the biochemical variables. In a study, the advantageous outcomes of mushrooms on the broilers’ growth performance and the protective antioxidant activity of tissue have been observed (Giannenas et al., 2010). Mushrooms possess significant quantity of oligosaccharides, which have useful influence on the broilers ‘performance (Falaki et al., 2011). Based on the research, it can be said that the combination of these two plants can have certain profit and can be used as an alternative to antibiotics. There are very few studies on the main and combined effects of these substances on the performance, meat quality, and serum biochemical parameters of broilers. Hence, this study was conducted to investigate the main and combined effects of garlic powder (GP) and mushroom powder (MP) on the performance, meat quality and, serum biochemical parameters of broiler chickens.

## 2 MATERIALS AND METHODS

### 2.1 Garlic and mushroom powder preparation and analysis

Fresh garlic and mushrooms were acquired from a native market (Rasht). Pristine garlic was peeled, crushed, and desiccated under sunlight at 30–35°C for 1 day (Lim et al., 2006). The arid herbs were dried (at 50°C) in an oven and ground into flour (Chowdhury et al., 2002). Mushrooms were also cut into slices with a thickness of 5 mm and dried at 55–60°C, and then ground to produce mushroom powder (Kavyani & Porreza, 2012). The dried GP and MP were analyzed to measure the quantity of dry matter (DM), crude protein (CP), crude fiber (CF), crude fat, and ash (AOAC, 2005). The data are presented in Table 1.

**TABLE 1:** Approximate analysis for dried garlic and mushroom powder.

Item	Garlic powder (GP)	Mushroom powder (MP)
Dry matter (%)	90.11	91.31
Crude protein (%)	11.94	12.51
Crude fibre (%)	1.63	6.41
Crude fat (%)	2.70	4.10
Ash (%)	3.15	7.61

### 2.2 Birds and management

Five hundred and seventy‐six one‐day‐old male Ross 308 broiler chicks were acquired from a commercial hatchery and reared for 42 days. Eight experimental mash diets (4*2, with six replicates) that were fed to the birds during the starter (1–10 d of age), grower (11–24 d of age), and finisher (25–42 d of age) phases included: (1) no GP + no MP (control), (2) 0.50% GP + no MP, (3) 1.00% GP + no MP, (4) 1.50% GP + no MP, (5) no GP + 1.00% MP, (6) 0.50% GP + 1.00% MP, (7) 1.00% GP + 1.00% MP, and (8) 1.50% GP + 1.00% MP. Feed additives were supplemented “on top” to the basal diets in each phase. The tables of the National Research Council were used for dietary nutrient composition (NRC, 1994). The ingredients and nutrient composition of diets are presented in Table 2. All diets were provided the nutrition specifications of Ross‐308 broilers (Aviagen, 2014). The temperature of the rearing site was scheduled at 32°C in the first 3 days and declined by 3°C each week to attain 21°C and remained stable till the ending of the trial. Relative humidity was between 60% and 70%. The first 2 days were set with a 24‐hour light schedule followed by 18 hours of light and 6 hours of darkness. The birds had access to feed and water within the whole study duration.

**TABLE 2:** Ingredients and nutrient composition of the experimental diets (as‐fed basis).

Ingredients (%)	Starter (1−10 d)	Grower (11−24 d)	Finisher (25−42 d)
Corn	52.39	55.75	61.02
Soybean meal (44%)	36.45	32.67	27.22
Corn gluten meal (62%)	3.00	3.00	3.00
Soybean oil	3.30	4.18	4.72
Limestone	1.08	0.99	0.91
Dicalcium phosphate	1.97	1.75	1.55
Common salt	0.26	0.26	0.26
Vitamin premix^a^	0.25	0.25	0.25
Mineral premix^b^	0.25	0.25	0.25
L‐Lysine HCl	0.37	0.30	0.29
DL‐Methionine	0.35	0.30	0.26
L‐Threonine	0.13	0.10	0.08
Sodium bicarbonate	0.16	0.16	0.16
Choline chloride 60%	0.04	0.04	0.04
Nutrient composition (%)			
Metabolizable energy (kcal/kg)	3000	3100	3200
Crude protein	23.00	21.50	19.50
Calcium	0.96	0.87	0.78
Available Phosphorus	0.48	0.43	0.39
Sodium	0.16	0.16	0.16
Lysine	1.44	1.29	1.15
Methionine + Cystine	1.08	0.99	0.90
Threonine	0.97	0.88	0.78

^a^ Provided the followings per kg of diet: vitamin A (trans‐retinyl acetate), 12,500 U; vitamin D3 (cholecalciferol), 5000 U; vitamin E (D L‐α tocopherol acetate), 80 U; vitamin K (menadione), 3.00 mg; riboflavin, 7.50 mg; pantothenic acid (D‐Ca pantothenate), 17.50 mg; pyridoxine (pyridoxine‐HCl), 4.50 mg; thiamine, 3.00 mg; vitamin B12 (cyanocobalamin), 0.02 mg; biotin, 0.20 mg; folic acid, 2.00 mg; nicotinic acid, 65.00 mg; ethoxyquin (antioxidant), 2.50 mg.

^b^ Provided the following per kg of diet: Fe, 20.00 mg; Zn, 110.00 mg; Mn, 120.00 mg; Cu, 16.00 mg; I, 1.25 mg; Se, 0.30 mg. The eight experimental treatments including: 1) no GP + no MP (control), 2) 0.50% GP + no MP, 3) 1.00% GP + no MP, 4) 1.50% GP + no MP, 5) no GP + 1.00% MP, 6) 0.50% GP + 1.00% MP, 7) 1.00% GP + 1.00% MP, and 8) 1.50% GP + 1.00% MP.) were added to basal diet during each phase.

### 2.3 Growth performance

The average body weight (ABW), daily weight gain (DWG), feed conversion ratio (FCR) and daily feed intake (DFI) were determined for each replicate (pen) at 42 d of age (Noruzi et al., 2022). Mortality was recorded daily and the viability of birds in each pen was monitored and noted. The European Production Efficiency Factor (EPEF) was measured with the formula:

$$\mathrm{EPEF}=\frac{\mathrm{Viability}\;\left(\%\right)\;\times \mathrm{body}\;\mathrm{weight}\;\left(\mathrm{kg}\right)}{\mathrm{age}\;\left(\mathrm{days}\right)\;\times \mathrm{FCR}\;\left(\mathrm{kg}\;\mathrm{feed}/\mathrm{kg}\;\mathrm{gain}\right)}\times 100$$

### 2.4 Meat quality

On day 42, two chickens, with an average body weight of each pen, were selected and euthanized by cervical dislocation. The right breast muscle was removed and kept at 4°C to measure meat quality. A CR410 Chroma meter (Konica Minolta Sensing Inc., Japan) was applied to investigate the muscle color indices (lightness L*, redness a*, and yellowness b*) 24 h after death (Zhang et al., 2011). In order to evaluate the pH of the muscles at 45 min and 24 h post‐mortem a WTW pH 330i pH Meter was used (Zhang et al., 2009). The cooked samples were analyzed for shear force using a Texture Analyzer (TA.XT plus, Stable Micro Systems) (Zhang et al., 2014).

### 2.5 Serum lipid profile

At 42 d of age, blood samples were gained via the wing vein from two birds in each pen. Blood samples were centrifuged (2000× g for 10 min) and serum was separated and stored at −20°C until the assayed for evaluating of the biochemical parameters. Appropriate laboratory kits (Pars Azmoon) determined triglycerides (Bucolo & David, 1973), total cholesterol (Zak et al., 1954), and high‐density lipoprotein cholesterol (HDL) (Naito & Kaplan, 1984). Very low‐density lipoprotein cholesterol (VLDL) was calculated by dividing triglyceride levels by 5, and low‐density lipoprotein cholesterol (LDL) was measured as the discrepancy amongst whole cholesterol and the composed condensations of HDL and VLDL (Friedewald et al., 1972).

### 2.6 The morphology of jejunum

Two chickens from every pen were randomly picked out and euthanized via translocation of cervical vertebrate at 42 d of age. The complete small intestine was picked up, and pieces of approximately 1 cm were removed from the middling of the jejunum. The pieces were stabilized in neutral buffered formalin solution (10%) and infixed in paraffin wax later. All analyses are accomplished on 5 µm portions and tainted with hematoxylin and eosin (Alshelmani et al., 2016a). A computer‐connected optical microscope (Olympus model BX51 microscope; magnification 100) was applied to acquire picture of the tissues, and consequently, the parameters including villus height (VH), villus width (VW), crypt depth (CD), and the thickness of muscle layer were measured using the relevant software (Garcia et al., 2007). Villus surface area was measured by (2л)*(VW/2)*(VH).

### 2.7 Statistical analysis

This study was done based on a completely randomized design (CRD) in a factorial arrangement of 4*2 with four levels of GP and two levels of MP with six replicates by the GLM^1^ procedure of SAS 9.4 software (SAS, 2012). As the main effect of GP (quantitative factor) was significant, regression analysis was done. When the interactions were significant, the regression analysis for the GP within each MP (qualitative factor) level was performed and their confidence intervals were compared. All data were normalized by the Shapiro–Wilk test before statistical analysis. The main effects were compared by Tukey's test (*p* < 0.05).

## 3 RESULTS

### 3.1 Growth performance

The results of ABW, DWG, DFI, FCR, and EPEF are illustrated in Table 3. The main and interaction effects of factors (GP and MP) were not significant (*p* > 0.05).

**TABLE 3:** Effects of different levels of dried garlic and mushroom powder on the performance of broilers chickens during the experimental period (1–42 d of age).

Treatments		Whole phase (1–42 d of age)			
Main effects	ABW (g)	DWG (g/bird/d)	DFI (g/bird/d)	FCR (g:g)	EPEF
GP (%)					
0.00	2377.33	55.62	85.90	1.54	351.45
0.50	2343.62	54.82	84.41	1.53	347.87
1.00	2359.45	55.20	85.13	1.54	348.42
1.50	2367.65	55.41	86.24	1.56	347.04
*p‐*value	0.51	0.52	0.61	0.91	0.94
SEM	16.203	0.385	1.038	0.018	5.204
MP (%)					
0.00	2355.64	55.11	85.27	1.55	347.64
1.00	2368.39	55.42	85.56	1.54	349.75
*p‐*value	0.44	0.43	0.78	0.88	0.68
SEM	11.457	0.272	0.734	0.013	3.679
Interaction effects					
No GP + no MP	2371.31	55.48	85.57	1.54	349.79
0.50% GP + no MP	2305.17	53.91	82.91	1.53	343.45
1.00% GP + no MP	2367.85	55.40	85.60	1.54	349.38
1.50% GP + no MP	2378.25	55.64	87.01	1.56	347.94
No GP + 1.00% MP	2383.36	55.77	86.24	1.55	353.12
0.50% GP + 1.00% MP	2382.08	55.74	85.89	1.54	352.30
1.00% GP + 1.00% MP	2351.09	55.00	84.67	1.54	347.47
1.50% GP + 1.00% MP	2357.05	55.15	85.44	1.55	346.13
*p‐*value	0.14	0.14	0.42	0.98	0.86
SEM	22.915	0.544	1.468	0.025	7.359

Abbreviations: ABW: average body weight; DWG: daily weight gain; DFI: daily feed intake; EPEF: European production efficiency factor; FCR: Feed conversion ratio; GP: garlic powder; MP: mushroom powder; SEM: Standard error of mean.

### 3.2 Meat quality

The data of meat quality at 42 d of age are shown in Table 4. The main effects of GP on the L* and a* of breast muscle were significant (*p* < 0.05). According to the regression equation (Table 5), with increasing levels of GP in the diets, the L* and a* of meat decreased and increased, respectively. The lack of fit for both of them was not significant (Table 5), showing that the data had a good fit with the model.

**TABLE 4:** Effects of different levels of dried garlic and mushroom powder on the breast muscle quality parameters of broilers at 42 d of age.

Treatments						
Main effects	Lightness	Redness	Yellowness	pH		Shear force
	(L^*^)	(a^*^)	(b^*^)	45 min	24 h	(N)
GP (%)						
0.00	53.93	6.91	16.64	6.11	5.78	29.28
0.50	53.34	7.21	16.90	6.18	5.93	29.19
1.00	52.61	7.84	16.64	6.24	5.81	28.93
1.50	52.55	7.92	17.00	6.17	5.86	28.98
*p‐*value	<0.0001	0.0002	0.28	0.81	0.32	0.14
SEM	0.127	0.168	0.157	0.092	0.059	0.117
MP (%)						
0.00	53.05	7.29	16.82	6.16	5.84	29.14
1.00	53.17	7.25	16.76	6.19	5.85	29.06
*p‐*value	0.34	0.33	0.68	0.75	0.96	0.52
SEM	0.090	0.102	0.111	0.065	0.042	0.082
Interaction effects						
No GP + no MP	54.14	6.61	16.87	6.03	5.69	29.31
0.50% GP + no MP	53.28	7.07	16.81	6.20	5.91	29.16
1.00% GP + no MP	52.52	7.62	16.65	6.31	5.86	28.99
1.50% GP + no MP	52.26	7.85	16.97	6.10	5.91	29.09
No GP + 1.00% MP	53.73	7.22	16.40	6.18	5.87	29.25
0.50% GP + 1.00% MP	53.41	7.35	16.99	6.15	5.95	29.22
1.00% GP + 1.00% MP	52.75	8.06	16.64	6.15	5.76	28.86
1.50% GP + 1.00% MP	52.80	7.99	17.01	6.26	5.80	28.91
*p‐*value	0.08	0.78	0.51	0.58	0.29	0.88
SEM	0.179	0.238	0.223	0.131	0.084	0.165

*Note*: *p* < 0.05: significant.

Abbreviations: GP: garlic powder; MP: mushroom powder; SEM: Standard error of mean.

**TABLE 5:** Regression analysis of the main effect of garlic powder (quantitative factor) on the lightness (L^*^) and redness (a^*^) of breast meat at 42 d of age.

		Lightness (L^*^)			Redness (a^*^)		
Source	df	Sum of squares	R^2^	*p*‐value	Sum of squares	R^2^	*p*‐value
Model	1	14.49	0.58	<0.0001	7.94	0.34	<0.0001
Lack of fit	2	1.02		0.1002	0.61		0.4307
Pure error	44	9.29			15.56		
Total error	46	10.32			16.17		
Equation		53.85 – 0.98GP			6.92 + 0.73GP		

*Note*: *p* < 0.05: significant.

Abbreviations: df: degrees of freedom; R^2^: coefficient of determination.

### 3.3 Serum lipid profile

The main and interaction effects of GP and MP on the serum lipid profile are shown in Table 6. The interaction effects of GP and MP on the serum total cholesterol level were significant (*p* < 0.05). Analysis of regression of the effects of GP in each level of MP for total cholesterol level is presented in Table 7. The regression equations showed that the effect of increasing the amount of GP on the reduction of total cholesterol level was similar in the absence or presence of MP. The lack of fit for both equations was insignificant, indicating that the data were in an appropriate agreement with their model (Table 7).

**TABLE 6:** Effects of different levels of dried garlic and mushroom powder on the serum lipid profile of broilers at 42 d of age.

Treatments				
Main effects	Total cholesterol (mg/dL)	HDL (mg/dL)	LDL (mg/dL)	VLDL (mg/dL)
GP (%)				
0.00	171.83	62.25	92.60	15.19
0.50	168.67	61.50	91.58	15.58
1.00	163.17	60.58	91.32	15.32
1.50	160.15	59.92	85.94	15.57
*p‐*value	0.002	0.64	0.19	0.59
SEM	2.159	1.361	2.49	0.241
MP (%)				
0.00	167.79	62.38	91.88	15.56
1.00	164.13	59.75	88.83	15.27
*p‐*value	0.10	0.06	0.20	0.23
SEM	1.527	0.963	1.671	0.171
Interaction effects				
No GP + no MP	174.31	61.67	97.13	15.53
0.50% GP + no MP	175.31	63.82	95.92	15.58
1.00% GP + no MP	159.00	61.16	90.63	15.32
1.50% GP + no MP	162.50	62.84	83.85	15.82
No GP + 1.00% MP	169.34	62.83	88.06	14.85
0.50% GP + 1.00% MP	162.01	59.17	87.25	15.58
1.00% GP + 1.00% MP	167.29	60.00	92.01	15.31
1.50% GP + 1.00% MP	157.80	57.01	88.00	15.33
*p‐*value	0.01	0.26	0.11	0.67
SEM	3.053	1.925	3.858	0.341

*Note*: *p* < 0.05: significant.

Abbreviations: GP: garlic powder; HDL: high‐density lipoprotein cholesterol; LDL: low‐density lipoprotein cholesterol; MP: mushroom powder; VLDL: Very low‐density lipoprotein cholesterol: SEM: Standard error of mean.

**TABLE 7:** Regression analysis of the effects of GP (quantitative factor) in each level of MP for total cholesterol at 42 d of age.

	Total cholesterol (mg/dL)						
	no MP				1.00% MP		
Source	df	Sum of squares	R^2^	P‐value	Sum of squares	R^2^	*p*‐value
Model	1	806.00	0.30	0.0053	255.21	0.20	0.0303
Lack of fit	2	423.78		0.0739	233.92		0.0799
Pure error	20	1424.17			813.50		
Total error	22	1847.95			1047.42		
Equations		175.57 – 10.37 GP			168.50 – 5.83 GP		
Confidence interval		(−17.31, −3.43)			(−11.06, −0.61)		

*Note*: *p* < 0.05: significant.

Abbreviations: df: degrees of freedom; R^2^: coefficient of determination; GP: garlic powder; MP: mushroom powder.

### 3.4 Intestinal morphology

The effects of GP and MP supplementation on the jejunum morphology characteristics of broilers are shown in Table 8. The width of villus, muscle thickness, and villus surface area were not affected by dietary treatments. The main effects of GP and MP on the VH and VH: CD of jejunum were significant (*p* < 0.05) (Table 8). Regarding the main effect of MP on the VH and VH: CD, the data showed that the use of MP in the diets significantly increased them (*p* < 0.05). According to the regression equation (Table 9), with increasing levels of GP in the diets, the VH and VH: CD increased. The non‐significance of lack of fit for both the VH and VH: CD models indicated the good agreement of the data with their models (Table 9).

**TABLE 8:** Effects of different levels of dried garlic and mushroom powder on the jejunum morphology at 42 d of age.

Experimental treatments	Jejunum morphology characteristics					
Main effects	Villus height (µm)	Villus width (µm)	Crypt depth (µm)	VH: CD	Muscle thickness (µm)	Villus surface area (mm^2^)
GP (%)						
0.00	1170.00	146.87	266.67	4.42	288.58	0.54
0.50	1188.08	148.36	266.17	4.51	267.33	0.56
1.00	1240.58	150.92	253.75	4.94	286.17	0.59
1.50	1237.76	150.98	256.34	5.07	289.67	0.59
*p‐*value	0.01	0.73	0.11	0.02	0.59	0.08
SEM	18.035	3.109	6.833	0.166	13.06	0.016
MP (%)						
0.00	1189.67b	147.52	263.13	4.56b	285.17	0.55
1.00	1228.54a	151.05	253.33	4.91a	280.71	0.58
*p‐*value	0.03	0.26	0.16	0.04	0.73	0.05
SEM	12.752	2.198	4.832	0.118	9.237	0.011
Interaction effects						
No GP + no MP	1146.33	144.34	270.67	4.26	268.84	0.52
0.50% GP + no MP	1175.50	147.40	268.65	4.42	260.85	0.55
1.00% GP + no MP	1219.69	148.17	264.82	4.63	308.50	0.57
1.50% GP + no MP	1217.17	150.17	248.30	4.95	302.50	0.57
No GP + 1.00% MP	1193.67	149.41	262.64	4.59	308.31	0.56
0.50% GP + 1.00% MP	1200.65	149.35	263.69	4.60	273.83	0.56
1.00% GP + 1.00% MP	1261.50	153.67	242.67	5.25	263.79	0.61
1.50% GP + 1.00% MP	1258.34	151.80	244.35	5.21	276.82	0.60
*p‐*value	0.97	0.95	0.77	0.81	0.12	0.93
SEM	25.505	4.592	9.664	0.235	18.474	0.023

*Note*: a and b show the means within a column without a common superscript significantly differ (*p* < 0.05).

Villus surface area was calculated by (2л)*(villus width/2)*(villus height).

Abbreviations: GP: garlic powder; MP: mushroom powder: VH: CD: villus height to crypt depth ratio; SEM: Standard error of mean.

**TABLE 9:** Regression analysis of the main effect of garlic powder (quantitative factor) on the villus height and villus height to crypt depth ratio of jejunum at 42 d of age.

		Villus height (VH)			Villus height to crypt depth ratio (VH:CD)		
Source	df	Sum of squares	R^2^	*p*‐value	Sum of squares	R^2^	p‐value
Model	1	39,245.00	0.18	0.0028	3.41	0.18	0.0025
Lack of fit	2	6145.57		0.4681	0.26		0.6826
Pure error	44	175,076.00			15.05		
Total error	46	181,222.00			15.31		
Equation		1170.74 + 51.15GP			4.38 + 0.48GP		

*Note*: *p* < 0.05: significant.

Abbreviations: df: degrees of freedom; R^2^: coefficient of determination.

## 4 DISCUSSION

Different levels of garlic and mushroom powder did not affect the performance of the birds during the experimental period. In line with the present study, some researchers expressed that the use of GP and garlic oil did not affect the birds’ performance (Chowdhury et al., 2002). Choi et al. (2010) and Reddy et al. (1991) did not observe any notable effect of GP and α‐tocopherol on the broilers’ performance within their period of study. Yalcin et al. (2007) reported that body weight, FI, and FCR were not affected by the dietary garlic powder in laying quails. Regarding the use of different levels of mushrooms in poultry diets, various studies have been conducted and different results have been obtained (Guimaraes et al., 2014; Shang et al., 2016). In accordance with our trial, Kavyani & Porreza (2012) observed that the inclusion of mushrooms did not affect the DWG and FCR within whole period of research. The reason for the variety of results can be quantities, species, application method (fermented or non‐fermented), plant parts used and treatment period (Mahfuz et al., 2020).

In the present trial, with increasing the levels of GP, the L* and a* of breast muscle reduced and increased, respectively, and the use of MP in the diets remarkably reduced the a* of meat samples. It has been stated that an important way to improve meat quality in poultry is diet manipulation (Abdulla et al., 2019). Parameters such as meat pH, tenderness, color (L*, a* and b*) and water holding capacity are related to specifications of meat quality (Mahfuz & Piao, 2019). There is a strong relationship between breast meat color and pH. A rapid decrease of muscle pH, while the carcass temperature is still high, may cause denaturation of the proteins in the muscles. Hence, the meat color becomes pale (Alshelmani et al., 2016b). Due to the high content of unsaturated fatty acids in poultry meat, the meat of this group of animals is easily oxidized by free radicals (Arshad et al., 2011). Xie et al. (2022) reported that antioxidation of olive leaf extract prevented the autoxidation of haem, leading to lowering the breast muscle lightness and enhancing the breast muscle redness. Fernandez‐Lopez et al. (2005) has also stated that the existence of antioxidant materials in herbal compounds can delay the constitution of methemoglobin in the muscles and therefore reduce L* values. It has been reported that garlic reduced L*, a*, and b* values in pork (Chen et al., 2008). It has been said that the antibacterial, anticoccidial, antifungal, antiviral, antioxidant activity, and immune‐enhancing activity of allium‐derived compounds improve the quality of poultry products (Kothari et al., 2019). Shang et al. (2014 (reported that the lightness and redness values for breast muscle were remarkably reduced with different Hericium caput‐medusae mushroom levels. Cho et al. (2014) declared that the use of phytogenic feed additive (thyme and star anise oils) in the broilers’ diet had no significant effect on the color of breast meat. Ding et al. (2020) did not observe significant differences in terms of pH, meat color, shear force and intramuscular fat of breast and thigh muscles when using different levels of star anise and its extractions in broilers’ diets.

Serum parameters are of diagnostic importance, have been explained to be main indexes of physiological, pathological, and nutritional situation of an organism, and could be applied to explicate the impacts of remedial or nutritional managements in human and veterinary medicine (Toghyani et al., 2012). In our study, the effect of increasing the GP level on the reduction of total cholesterol level was similar in the absence or presence of MP. This means that increased levels of GP can lower cholesterol on its own. It has been proven that garlic is a plant that has remedial efficacy such as reducing total cholesterol of serum (Lawrence & Lawrence, 2011). Qureshi et al. (1983) reported that the activities of hydroxymethylglutaryl coenzyme A reductase, cholesterol 7α‐hydroxylase and fatty acid synthetase decreased in birds consuming GP. Some researchers attributed the effect of garlic on the reducing serum cholesterol to the sulfur compounds present in it, especially S‐Allyl cysteine (Windisch et al., 2008). Onyimonyi et al. (2012) observed that the addition of GP (0.75%) to the broilers’ diet reduced the total cholesterol amount remarkably when contrasted to the other treatments (0.25% and 0.50). It has been reported that the use of 3% GP in the diet of broiler chickens led to a decrease in cholesterol levels in plasma, liver, breast and thigh muscles (Konjufca et al., 1997). Ismail et al. (2021) also found that adding different levels of garlic powder (0.25, 0.50, and 0.75 g/kg) to the diet of broilers significantly reduced serum cholesterol compared to the control treatment. In contrast, Lim et al. (2006) observed that the amount of HDL was not affected by feeding of GP (0, 1, 3, and 5%) in layers. In accordance with our experiment, Daneshmand et al. (2011) reported that the use of 2 g/kg of oyster mushroom in the diet of broilers did not have a significant effect on reducing serum cholesterol.

In the current study, a significant increase of VH and CH: CD of the jejunum was noticed, so that with increasing the level of GP in the diets, the levels of these two parameters increased. In addition, the chickens receiving the MP showed higher VH and VH:CD. The safety and health of the gastrointestinal tract and improved absorption ability of the intestines were ascribed to the higher VH and lower CD (Hassan et al., 2020). The enhancement of VH improves the absorption of nutrients because of higher surface area, while deeper crypt shows increment cell turnover in response to normal or inflammatory condition. The antioxidant attributes of GP may ameliorate growth and development of the birds’ digestive tract (Halliwell et al., 2000). On the other hand, due to the antibacterial virtues of garlic, it can reduce the population of harmful bacteria in the digestive tract and thus improve its physiological condition (Jimoh et al., 2013). In agreement with our study, Lee et al. (2016) found that the VH: CD in jejunum of broilers linearly enhanced with increasing fermented garlic levels. Considering that the VH:CD indicates the digestive capacity of the small intestine, an increment in this ratio indicate that garlic may play a role in digestion and absorption, as reported by other researchers (Adibmoradi et al., 2006). Gross & Siegel (1983) reported that birds fed 1.00 and 2.00% oyster mushroom waste had an increase in VH in the duodenum, ileum, and jejunum. The possible reason is the high fiber content of the mushroom, which leads to structural changes in the small intestine of the bird. Shang et al. (2016) reported that intestinal VH and VH: CD were significantly higher in mushroom‐fed birds than the control groups. Giannenas et al. (2011) reported that increased VH was observed in the duodenum, jejunum and ileum of birds fed with 1.00 and 2.00% mushrooms, which was consistent with the results of the present experiment.

## 5 CONCLUSIONS

The outcomes revealed that the addition of garlic and mushroom powder to the broilers’ diets did not have any negative effect on the growth performance. By increasing the level of garlic powder in the diet, it was observed that the meat color and intestinal morphology improved and the serum cholesterol level decreased. The presence of mushroom powder in the diets could increase the height of the villus and villus height to crypt depth ratio.

## AUTHOR CONTRIBUTIONS

**Fatemeh Aziz‐Aliabadi**: Conceptualization; Formal analysis; Funding acquisition; Investigation; Methodology; Project administration; Resources; Software; Writing—original draft; Writing—review and editing.

**Hadi Noruzi**: Conceptualization; Formal analysis; Data curation; Funding acquisition; Investigation; Methodology; Project administration; Resources; Software; Writing—original draft; Writing—review and editing.

**Zeyad Kamal Imari**: Conceptualization; Investigation; Software; Writing—review & editing.

## CONFLICT OF INTEREST STATEMENT

The authors declare no conflicts of interest.

## FUNDING INFORMATION

All the costs of the experiment were covered by the authors' personal funds.

## ETHICS STATEMENT

All procedures were approved by the Animal Care and Use Committee of the Ferdowsi University of Mashhad, Mashhad, Iran.

### PEER REVIEW

The peer review history for this article is available at https://publons.com/publon/10.1002/vms3.70031.

## Data Availability

The data that support this study will be shared upon reasonable request to the corresponding author.

## Footnotes

1 General linear model
